# CEACAM6 is a determinant of pancreatic adenocarcinoma cellular invasiveness

**DOI:** 10.1038/sj.bjc.6602113

**Published:** 2004-08-17

**Authors:** M S Duxbury, H Ito, E Benoit, S W Ashley, E E Whang

**Affiliations:** 1 Department of Surgery, Brigham and Women's Hospital, Harvard Medical School, 75 Francis Street, Boston, MA 02115, USA

**Keywords:** pancreatic cancer, adenocarcinoma, carcinoembryonic antigen-related cell adhesion molecule 6, siRNA, invasion, MMP-9

## Abstract

Pancreatic adenocarcinoma is among the most aggressively invasive malignancies. The immunoglobulin superfamily member carcinoembryonic antigen-related cell adhesion molecule 6 (CEACAM6) is emerging as an important determinant of the malignant phenotype in a range of cancers. We sought to define the role of CEACAM6 in pancreatic adenocarcinoma cellular invasiveness. CEACAM6 was stably overexpressed in Capan2 cells, which inherently express low levels of CEACAM6. Retrovirally mediated RNA interference was used to silence CEACAM6 expression in BxPC3 cells, which inherently overexpress CEACAM6. Cellular invasiveness was quantified using a modified Boyden chamber assay. Overexpression of CEACAM6 increased Capan2 cellular invasiveness, whereas CEACAM6 knockdown attenuated BxPC3 invasiveness. A role for the c-Src tyrosine kinase in mediating CEACAM6-dependent invasiveness was defined using constitutively active and dominant-negative c-Src expression constructs. c-Src-dependent modulation of matrix metalloproteinase-9 activity contributes significantly to the increased cellular invasiveness induced by CEACAM6 overexpression. Levels of CEACAM6 expression can modulate pancreatic adenocarcinoma cellular invasiveness in a c-Src-dependent manner. This pathway warrants further investigation as a target for therapy.

The outlook for those with pancreatic adenocarcinoma has improved little over the past decade, despite significant advances in the perioperative management of patients with this disease (Postier, 2003). Among the most significant determinants of the dismal prognosis associated with this malignancy are the highly aggressive loco-regional invasion and early metastasis that characterise this malignancy, such that the majority of patients present with advanced, surgically unresectable disease (Brand, 2001; Tamm and Charnsangavej, 2001). Novel therapeutic approaches to this malignancy are urgently needed.

Carcinoembryonic antigen-related cell adhesion molecule 6 (CEACAM6) is a glycosylphosphatidylinositol (GPI)-linked immunoglobulin superfamily member that is overexpressed in a variety of gastrointestinal malignancies (Kodera *et al*, 1993; Jantscheff *et al*, 2003). Despite lacking a transmembrane or intracellular domain, CEACAM6 is able to influence intracellular signalling events and plays an important role in gastrointestinal cancer progression (Hasegawa *et al*, 1993; Kodera *et al*, 1993; Scholzel *et al*, 2000; Ilantzis *et al*, 2002). Pancreatic adenocarcinoma cells differentially express CEACAM6 and overexpression of CEACAM6 is associated with greater resistance to anoikis, a subset of apoptosis induced by inadequate or inappropriate cell substrate adhesion (Gardner-Thorpe *et al*, 2002; Duxbury *et al*, 2004b). Resistance to anoikis is a property of transformed cells that is associated with greater cellular invasive ability and *in vivo* metastatic potential (Yawata *et al*, 1998; Streuli and Gilmore, 1999; Shanmugathasan and Jothy, 2000; Zhu *et al*, 2001). CEACAM6 overexpression is associated with greater resistance to anoikis and increased metastatic potential, whereas post-transcriptional inhibition of CEACAM6 expression impairs the ability of pancreatic adenocarcinoma cells to resist anoikis and to form experimental liver metastases *in vivo* (Duxbury *et al*, 2004b). These features of CEACAM6 make it a rational target for therapeutic intervention. Despite considerable evidence implicating CEACAM6 as an important determinant of the malignant phenotype, its role in cellular invasiveness has not been examined.

Here, we characterise for the first time the role of CEACAM6, and its downstream signalling targets, in determining pancreatic adenocarcinoma cellular invasiveness. Our results indicate that levels of CEACAM6 expression modulate pancreatic adenocarcinoma cellular invasiveness.

## MATERIALS AND METHODS

### Cells and cell culture

Capan2 and BxPC3 cells were obtained from the American Type Culture Collection (ATCC, Rockville, MD, USA). The Capan2 cell line is derived from a well-differentiated human pancreatic ductal adenocarcinoma. BxPC3 is derived from a moderately differentiated human pancreatic ductal adenocarcinoma (Berrozpe *et al*, 1994; Sipos *et al*, 2003). Cells were maintained in Dulbecco's modified Eagle medium (DMEM) containing 10% foetal bovine serum (FBS, Gibco BRL, Gaithersburg, MD, USA), and were incubated in a humidified (37°C, 5% CO_2_) incubator, grown in 75 cm^2^ culture flasks and passaged upon reaching 80% confluence.

### Expression vector construction and transfection

Poly-A RNA was reverse transcribed using an anchored oligo-dT primer with an *Xho*I restriction site. Double-stranded CEACAM6 cDNA was prepared and ligated into *Eco*RI adaptors, digested with *Xho*I, ligated into the pOTB7 vector and transferred to the pIRES expression plasmid (termed pIRES-CEACAM6). Cells were transfected with pIRES-CEACAM6 or pIRES-eGFP, which acted as a control, using Lipofectamine 2000 (Invitrogen, Carlsbad, CA, USA) in accordance with the manufacturer's protocol. Stable clones were selected by continuous treatment with G418 (Gibco, 0.8 mg ml^−1^). Constitutively active (myristoylated) Akt (myr-Akt), constitutively active Src (SrcY529F) and dominant-negative Src (Src(K296R/Y528F)) expression constructs were obtained from Upstate (Waltham, MA, USA). Transient transfection was performed using Lipofectamine 2000.

### Retrovirally mediated CEACAM6 RNA interference (RNAi)

CEACAM6-specific and single base mismatch small-interfering RNA (siRNA) expression constructs were generated by ligation of inserts (containing *Xho*I and *Xba*I overhang sites) targeting the following sequences into the pSuppressorNeo expression vector in accordance with the manufacturer's instructions (Imgenex, San Diego, CA, USA): CEACAM6 (psiCEACAM6): 5′-CCGGACAGTTCCATGTATA-3′; Control (psiControl): 5′-CCCGACAGTTCCATGTATA-3′. Correct insert ligation was confirmed by absence of linearisation by *Sal*I. Vectors were expanded in competent *Escherichia* coli. 293 packaging cells (ATCC) were transfected with retroviral vectors, using Lipofectamine 2000 (Invitrogen, Carlsbad, CA, USA), and selected for stable retrovirus production using G418. Pancreatic adenocarcinoma cells were exposed to retrovirus-containing supernatant for 16 h in the presence of polybrene (Sigma, St Louis, MO, USA). Stable selection of transfected clones was achieved using G418. Clones were maintained in 500 *μ*M G418.

### Western blotting

Cells were harvested and rinsed twice with PBS. Cell extracts were prepared with lysis buffer (20 mM Tris, pH 7.5, 0.1% Triton X, 0.5% deoxycholate, 1 mM PMSF, 10 *μ*g ml^−1^ aprotinin, 10 *μ*g ml^−1^ leupeptin) and cleared by centrifugation at 12 000 **g**, 4°C. Total protein concentration was measured using the BCA assay kit (Sigma, St Louis, MO, USA) with bovine serum albumin as a standard, according to the manufacturer's instructions. Cell extracts containing 30 *μ*g total protein were subjected to 10% SDS/PAGE, and the resolved proteins transferred electrophoretically to PVDF membranes (Invitrogen, Carlsbad, CA, USA). Equal protein loading was confirmed by Coomassie (BioRad, Hercules, CA, USA) staining of the gel. After blocking with PBS containing 0.2% casein for 1 h at room temperature, membranes were incubated with 3–5 *μ*g ml^−1^ antibody in PBS containing 0.1% Tween 20 overnight at 4°C. Anti-CEACAM6 and anti-CEA monoclonal antibodies were obtained from InnoGenex (San Ramon, CA, USA), anti-c-Src monoclonal antibody was obtained from Upstate Biotechnology (Lake Placid, NY, USA). Chemoluminescent detection (Upstate, Lake Placid, NY, USA) was performed in accordance with the manufacturer's instructions. The CEACAM6 signal was quantified using ImagePro Plus software version 4.0 and normalised to that of actin. Blots were performed in triplicate. Mean densitometric values (±s.d.) are shown.

### c-Src tyrosine kinase assay

c-Src tyrosine kinase activity was determined using a commercially available kinase assay kit (Sigma, St Louis, MO, USA), according to the manufacturer's instructions. c-Src immunoprecipitates (20 *μ*g total protein) were prepared using anti-c-Src monoclonal antibody immobilised onto protein G sepharose beads (Zymed Laboratories Inc., San Francisco, CA, USA). Immunoprecipitates were washed and dissolved in tyrosine kinase buffer (final solution containing 0.3 mM ATP) and incubated for 30 min in 96-well plates coated with tyrosine kinase substrate solution (poly-Glu-Tyr). Phosphorylated substrate was quantified by chromogenic detection using horseradish peroxidase-conjugated anti-phosphotyrosine antibody. Optical densities were determined at 492 nm using a *V*_max_ microplate spectrophotometer. c-Src kinase activity was compared to an epidermal growth factor receptor standard. Kinase assays were performed in triplicate with four determinations per condition.

### Invasion assay

Cellular invasion was quantified using a modified Matrigel Boyden chamber assay. The BD BioCoat Matrigel invasion chamber (BD Bioscience, Bedford, MA, USA) was used according to the manufacturer's instructions. In all, 2.5 × 10^4^ pancreatic cancer cells in serum-free media were seeded onto Matrigel-coated filters. In the lower chambers, 5% FBS was added as a chemoattractant. After 24 h incubation, the filters were stained with Diff-Quik™ kit (BD Biosciences), and the number of cells that had invaded through the filter was counted under magnification (randomly selected high-power fields). The counting was performed for three fields in each sample, and mean values from three independent experiments were calculated. The role of MMP-9 was determined by performing invasion assays in the presence of 10 *μ*g ml^−1^ anti-MMP-9 neutralising antibody or control (irrelevant) immunoglobulin (IgG).

### MMP-9 activity assay

MMP-9 activities of cell lysates were assessed using the colorimetric Biotrak MMP-9 activity assay (Amersham, Piscataway, NJ, USA), in accordance with the manufacturer's instructions. Optical densities were quantified using a *V*_max_ microplate spectrophotometer at a wavelength of 405 nm, referenced to 650 nm. Three samples were used for each experimental condition. Experiments were performed in triplicate and mean values calculated.

### Nude mouse xenograft model

Male athymic nu/nu mice, 5 weeks of age, weighing 20–22 g and specific pathogen-free, were obtained from Charles River Laboratories (Wilmington, MA, USA). Mice were housed in microisolator cages with autoclaved bedding in a specific pathogen-free facility with 12 h light–dark cycles and were maintained in accordance with the guidelines of the Harvard Medical Area Standing Committee on Animals and with the United Kingdom Co-ordinating Committee on Cancer Research (UKCCCR) Guidelines for the Welfare of Animals in Experimental Neoplasia (Workman *et al*, 1998). Mice were anaesthetised with intraperitoneal ketamine (200 mg kg^−1^), xylazine (10 mg kg^−1^) and xenografted with 10^6^ pancreatic adenocarcinoma cells in 75 *μ*l PBS by subcutaneous implantation. In all, 10 mice received psiCEACAM6 transfectants and 10 mice received psiControl transfectants. After 6 weeks, necropsy was performed and tumours were excised. Levels of CEACAM6 expression and MMP-9 activities of tumour lysates were determined by Western blotting (relative to actin) and Biotrak assay (controlling for total protein), respectively.

### Statistical analysis

Differences between groups were analysed using Student's *t*-test, multifactorial ANOVA of initial measurements and Mann–Whitney *U*-test, for nonparametric data, as appropriate, using Statistica 5.5. software (StatSoft, Inc, Tulsa, OK, USA). In cases in which averages were normalised to controls, the standard deviations of each nominator and denominator were taken into account in calculating the final standard deviation. *P*<0.05 was considered statistically significant.

## RESULTS

### Overexpression of CEACAM6 increases Capan2 pancreatic adenocarcinoma cellular invasiveness

**Figure 1:**
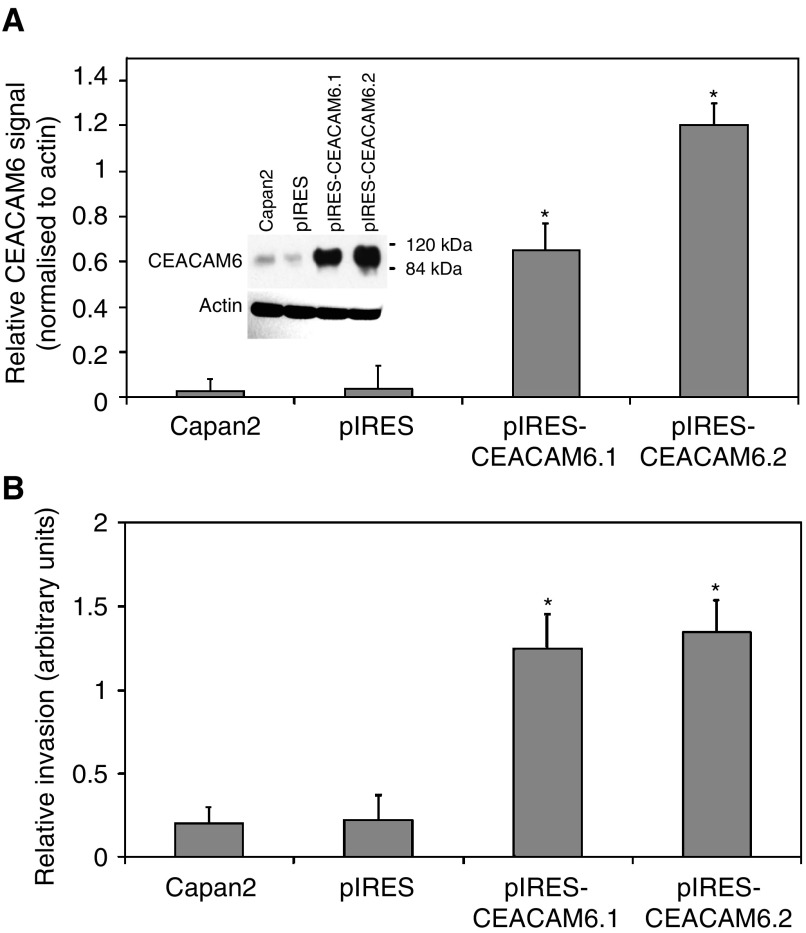
(**A**) Stable overexpression of CEACAM6 was confirmed in two Capan2-derived transfectants, pIRES-CEACAM6.1 and pIRES-CEACAM6.2, by Western blotting. Representative example with mean densitometric values (±s.d.) from triplicate blots. ^*^P<0.05 *vs* pIRES (empty vector) transfectants. (**B**) Cellular invasiveness in the Matrigel Boyden chamber assay was significantly increased in both pIRES-CEACAM6.1 and pIRES-CEACAM6.2 transfectants. Values are means (±s.d.) from triplicate experiments. ^*^P<0.05 *vs* pIRES transfectants.

CEACAM6 overexpression was induced in Capan2 cells, which inherently express a very low level of this molecule (Gardner-Thorpe *et al*, 2002; Duxbury *et al*, 2004b). Two stable CEACAM6-overexpressing clones (pIRES-CEACAM6.1 and pIRES-CEACAM6.2) were established and overexpression of CEACAM6 was confirmed by Western blot analysis (Figure 1A

). CEACAM6 expression in pIRES-CEACAM6.1 and pIRES-CEACAM6.2 transfectants was 16- and 30-fold that of control cells, respectively. Empty vector (pIRES) transfectants exhibited no change in CEACAM6 expression, relative to parental Capan2 cells. Cellular invasiveness towards a 5% FBS chemoattractant was quantified using a modified Matrigel Boyden chamber assay. pIRES transfectants and mock-transfected cells (Capan2) served as controls and did not differ in their cellular invasiveness. The cellular invasiveness of pIRES-CEACAM6.1 was 5.7-fold that of control, pIRES-CEACAM6.2 invasiveness was six-fold that of control (Figure 1B).

### Retrovirally mediated RNAi specific for CEACAM6 suppresses CEACAM6 expression

**Figure 2:**
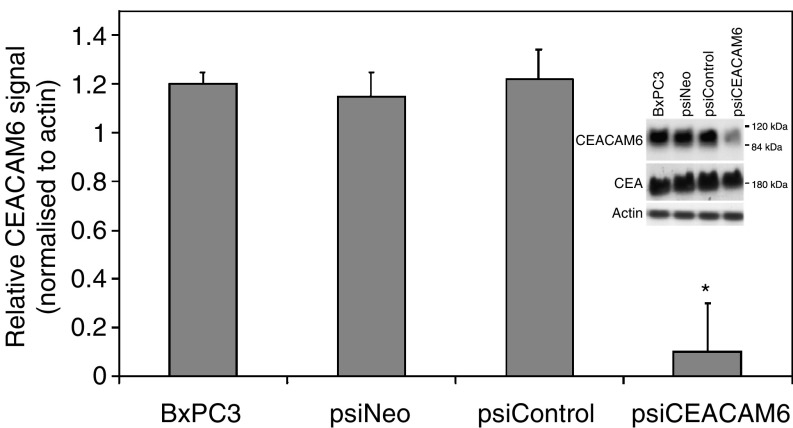
Stable suppression of CEACAM6 expression was demonstrated in BxPC3 psiCEACAM6 transfectants, by Western blot analysis. CEA expression did not significantly differ among the transfectants. Representative example with mean densitometric values (±s.d.) from triplicate blots. ^*^P<0.05 *vs* both psiNeo (empty vector) and psiControl transfectants.

BxPC3 cells were used to assess the potential utility of retrovirally mediated RNAi as an approach to silence CEACAM6 expression in pancreatic adenocarcinoma cells. This cell line was selected as it inherently expresses high levels of CEACAM6 (Gardner-Thorpe *et al*, 2002; Duxbury *et al*, 2004b). RNA interference induced by a siRNA-generating retroviral vector was used to suppress CEACAM6 expression. Total levels of CEACAM6 expression, determined by Western blot, were reduced by over 80% in BxPC3 cells stably transfected with the CEACAM6-specific siRNA-generating retroviral vector (psiCEACAM6). CEACAM6 expression was unaffected by the control retrovirus (psiNeo), relative to uninfected cells (Figure 2

). The specificity of the retrovirus was demonstrated by Western blotting for the closely related CEACAM family member, CEA. Levels of CEA expression were unaffected by either retrovirus.

### Retrovirally mediated RNAi specific for CEACAM6 attenuates BxPC3 pancreatic adenocarcinoma cellular invasiveness

**Figure 3:**
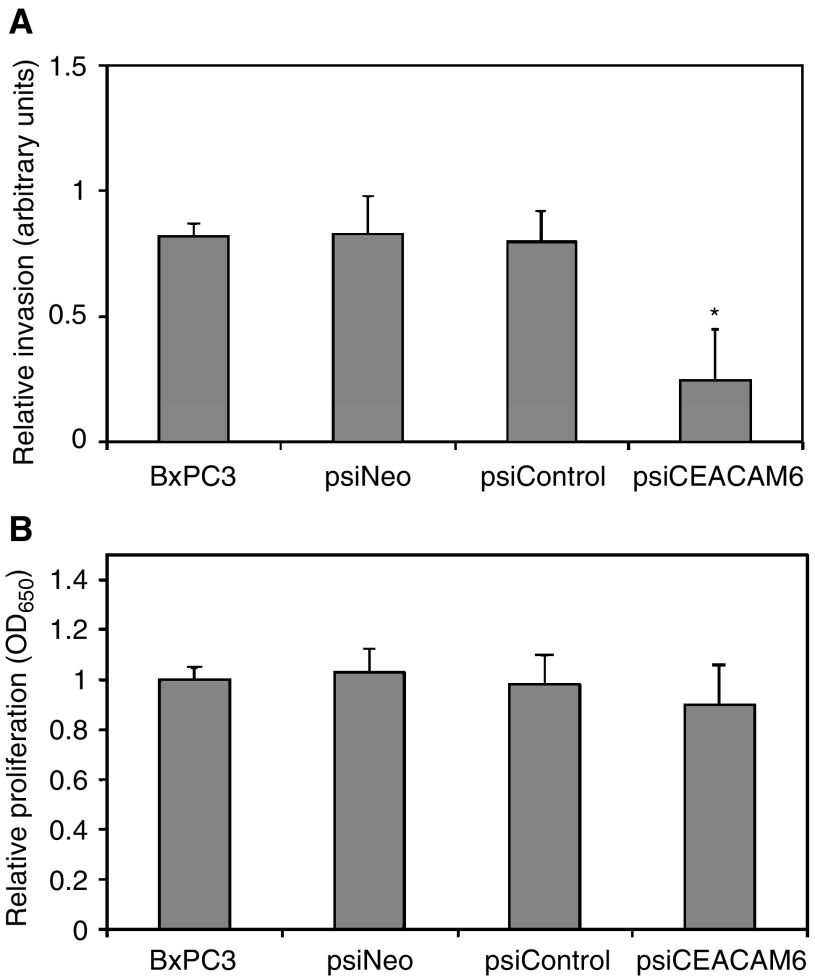
(**A**) Cellular invasiveness of psiCEACAM6 transfectants in the Matrigel Boyden chamber assay was significantly reduced, relative to psiNeo and psiControl transfectants. Values are means (±s.d.) from triplicate experiments. ^*^P<0.05 *vs* both psiNeo and psiControl transfectants. (**B**) Cellular proliferation of psiCEACAM6 transfectants, quantified by MTT assay, did not significantly differ from that of psiNeo or psiControl transfectants. Values are means (±s.d.) from triplicate experiments.

To assess the functional consequences of suppressing CEACAM6 expression, we quantified the ability of psiCEACAM6 transfectants to invade through a Matrigel simulated basement membrane in a modified Boyden chamber, relative to psiNeo transfectants and untreated BxPC3 cells. The cellular invasiveness of psiCEACAM6 transfectants through Matrigel towards a 5% FBS chemoattractant was 3.2-fold less than that of both psiNeo transfectants and untreated BxPC3 cells (Figure 3A

). Cellular invasiveness of psiNeo transfectants did not significantly differ from that of untreated BxPC3 cells. Although we have previously shown that modulation of CEACAM6 expression does not affect cellular proliferation of pancreatic adenocarcinoma cells (Duxbury *et al*, 2004b), it was important to determine the effect of retroviral infection on BxPC3 cellular proliferation. Proliferation of untreated cells, as well as cells exposed to CEACAM6-specific or control retrovirus, was quantified by MTT assay. We observed no significant difference in cellular proliferation rate among psiCEACAM6 and psiNeo transfectants, indicating not only that CEACAM6 expression is not essential for cellular survival and proliferation, but also that retroviral infection does not induce significant cytotoxicity (Figure 3B).

### Roles for c-Src and MMP-9 in CEACAM6-dependent cellular invasiveness

**Figure 4:**
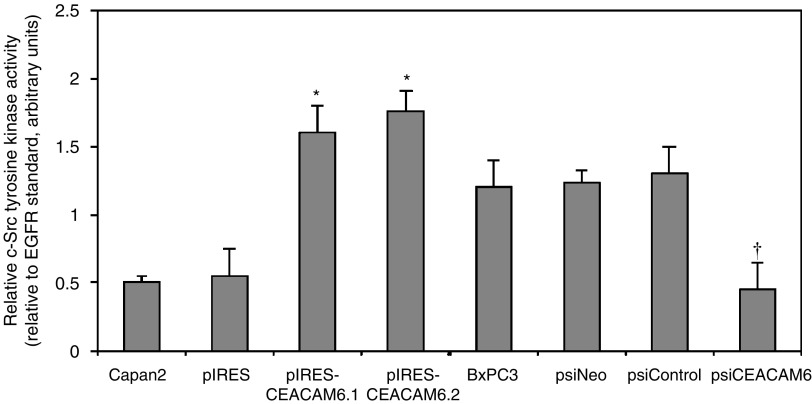
Both pIRES-CEACAM6.1 and pIRES-CEACAM6.2 Capan2-derived transfectants exhibited increased c-Src kinase activity, relative to pIRES transfectants. Conversely, suppression of CEACAM6 expression was associated with decreased c-Src kinase activity in BxPC3-derived psiCEACAM6 transfectants. Values are means (±s.d.) from triplicate experiments. ^*^P<0.05 *vs* pIRES transfectants. ^†^*P*<0.05 *vs* psiControl and psiNeo transfectants.

Src kinases are known to associate with GPI-anchored proteins, including those of the CEACAM family, and appear to play an important role in transducing downstream signalling events (Stefanova *et al*, 1991; Brown, 1993; Skubitz *et al*, 1995). We have previously shown that the Src family kinase inhibitor pyrazolopyrimidine (PP2) inhibits pancreatic adenocarcinoma cellular invasiveness in association with dephosphorylation of the prototype Src family kinase, c-Src, and suppression of MMP-2 and MMP-9 activities (Ito *et al*, 2003). Further evidence supports an important role for c-Src in modulating expression of MMP-9, in particular, in human cancer cells (Sato and Seiki, 1993; Recchia *et al*, 2003). We speculated that c-Src may play a role in the CEACAM6-dependent component of the invasive cellular phenotype and that this may occur through modulation of MMP-9 expression. In order to test this hypothesis, we first examined c-Src kinase activity following overexpression of CEACAM6, using a c-Src-specific *in vitro* kinase assay. Next, we examined the effect of retroviral CEACAM6-specific RNAi on c-Src activity. Both pIRES-CEACAM6.1 and pIRES-CEACAM6.2 transfectants exhibited increased cellular c-Src activities, relative to control transfectants. Conversely, suppression of CEACAM6 expression was associated with a significant decrease in c-Src activity (Figure 4

).

**Figure 5:**
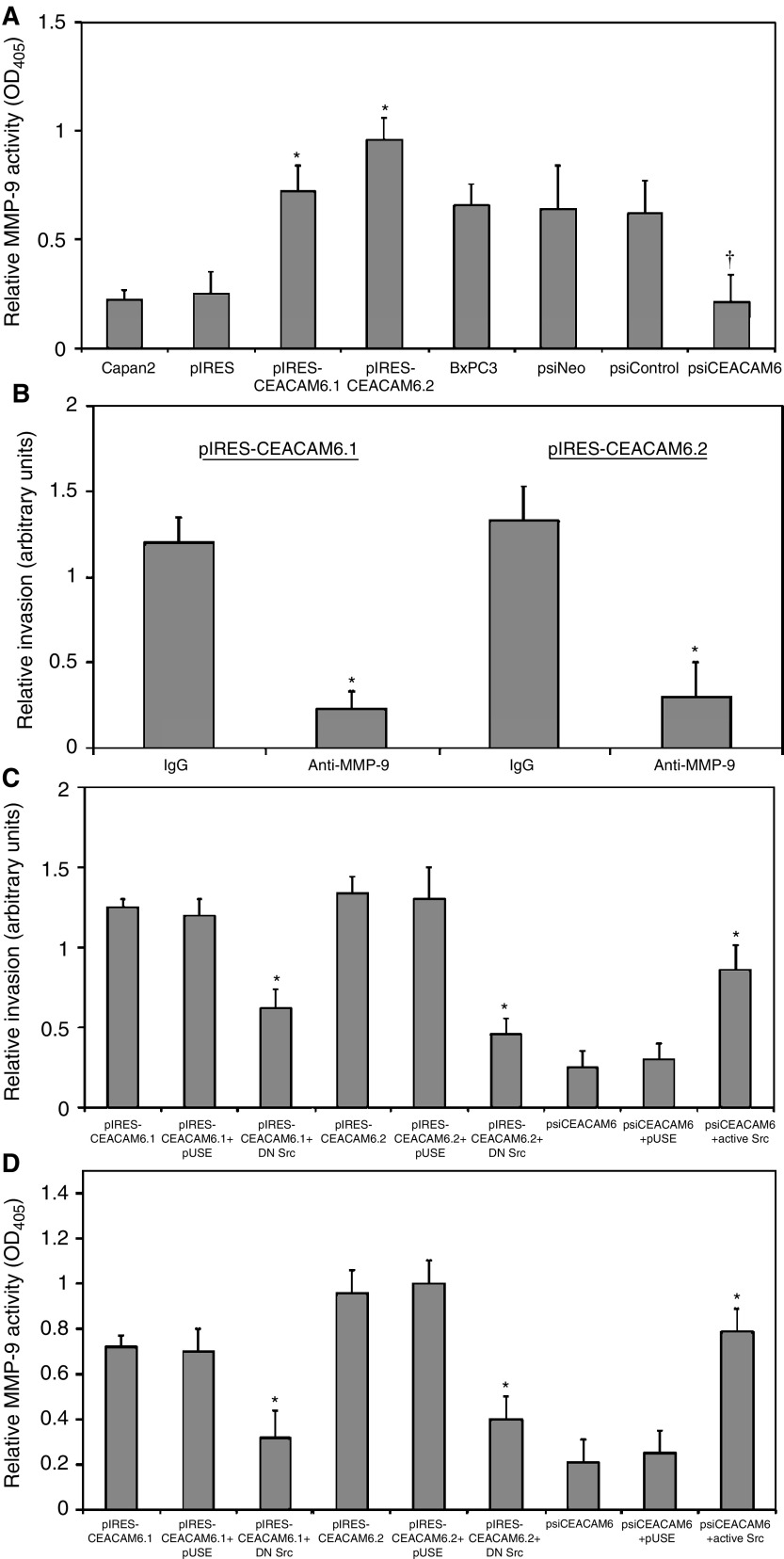
(**A**) MMP-9 activities were significantly increased in pIRES-CEACAM6.1 and pIRES-CEACAM6.2 transfectants, relative to pIRES transfectants. psiCEACAM6 transfectants exhibited significantly lower MMP-9 activity than both psiNeo and psiControl transfectants. Mean (±s.d.) from triplicate experiments. ^*^P<0.05 *vs* respective control transfectants. (**B**) Cellular invasiveness of pIRES-CEACAM6.1 and pIRES-CEACAM6.2 transfectants was significantly attenuated by exposure to anti-MMP-9 monoclonal antibody, but not isotype-matched irrelevant IgG. Mean (±s.d.) from triplicate experiments. ^*^P<0.05 *vs* control IgG. Overexpression of dominant-negative Src (DN Src) significantly decreased cellular invasiveness (**C**) and MMP-9 activities (**D**) of pIRES-CEACAM6.1 and pIRES-CEACAM6.2 transfectants. Constitutively active c-Src induced a significant recovery of MMP-9 activity (**C**) and cellular invasiveness (**D**) in psiCEACAM6 transfectants. Values are means (±s.d.) from triplicate experiments. ^*^P<0.05 *vs* pUSE empty vector.

Cellular MMP-9 activity was assessed by Biotrak assay. Both pIRES-CEACAM6.1 and pIRES-CEACAM6.2 transfectants exhibit significantly higher MMP-9 activities than both parental Capan2 cells and empty vector transfectants (Figure 5A

). Furthermore, addition of neutralising MMP-9 antibody, but not control isotype-matched irrelevant IgG, attenuated the cellular invasiveness of CEACAM6.1 and pIRES-CEACAM6.2 transfectants by 81 and 77%, respectively (Figure 5B). Inhibition of c-Src activity using a dominant-negative c-Src construct suppressed pIRES-CEACAM6.1 and pIRES-CEACAM6.2 cellular invasiveness by 48 and 65%, respectively (Figure 5C), and almost completely abolished the elevated MMP-9 activities exhibited by these transfectants (Figure 5D).

In order to examine the role of c-Src in mediating the effects of CEACAM6 on the invasive phenotype further, we tested the effects of expression of constitutively active c-Src on psiCEACAM6 transfectants. In keeping with our previous findings, introduction of constitutively active c-Src was sufficient to induce significant recovery of cellular invasiveness and MMP-9 secretion in psiCEACAM6 transfectants (Figure 5C and D). These observations are consistent with a model in which CEACAM6 overexpression increases cellular invasiveness, at least in part via a c-Src-dependent increase in MMP-9 activity.

### Effect of retrovirally mediated CEACAM6 knockdown on *in vivo* MMP-9 activity

**Figure 6:**
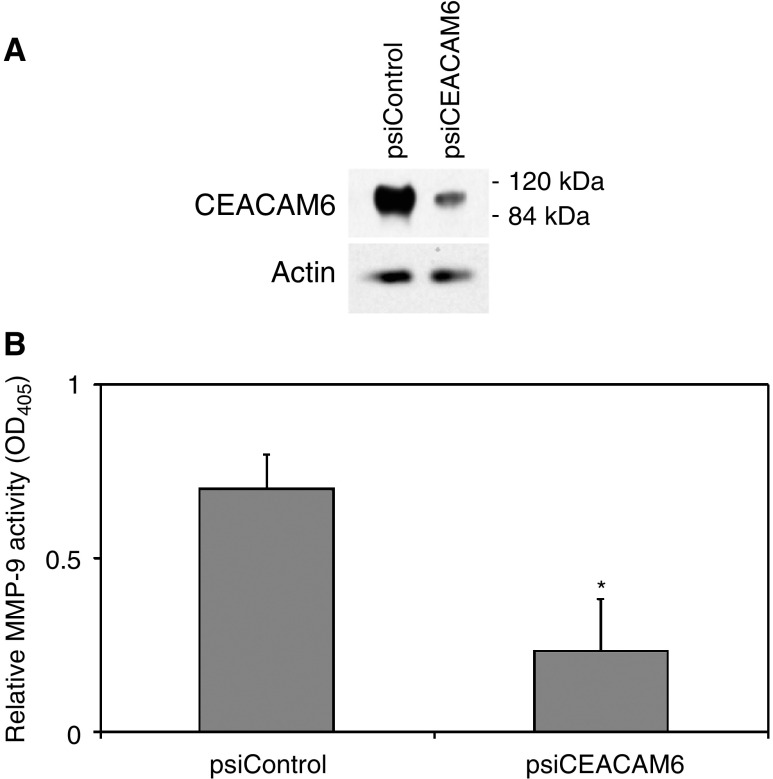
(**A**) Sustained knockdown of CEACAM6 expression was confirmed in the excised tumours by Western blot analysis. A representative blot is shown. (**B**) MMP-9 activities in lysates obtained from psiCEACAM6 transfectants were significantly lower than those of psiControl-derived tumours. Mean values (±s.d.). ^*^P<0.05 *vs* psiControl transfectant tumours.

Mice were subcutaneously xenografted with either BxPC3 psiCEACAM6 or psiControl transfectants. After 6 weeks, necropsy was performed and tumour lysates prepared. Western blot analysis confirmed lower levels of CEACAM6 expression in lysates from psiCEACAM6-derived tumours, relative to those derived from psiControl transfectants (Figure 6A

). The mean MMP-9 activities of lysates extracted from psiCEACAM6-derived tumours were significantly lower than those of psiControl-derived tumours (Figure 6B).

## DISCUSSION

Pancreatic adenocarcinoma is among the most invasive of all malignancies and locoregional tumour invasion is a common cause of inoperability. The purpose of this study was to define the role of CEACAM6 in determining the invasive cellular phenotype. We have shown that overexpression of CEACAM6 enhances cellular invasiveness and that post-transcriptional suppression of CEACAM6 expression decreases the invasiveness of pancreatic adenocarcinoma cells. CEACAM6 appears to exert its pro-invasive effect in a c-Src-dependent manner, at least in part through upregulation of MMP-9 activity.

Increasing evidence implicates CEACAM6 as a determinant of malignant cellular behaviour and clinical outcome in a range of human cancers (Hasegawa *et al*, 1993; Kodera *et al*, 1993; Scholzel *et al*, 2000; Jantscheff *et al*, 2003). While CEACAM6 is recognised to act as an intercellular adhesion molecule (Kuroki *et al*, 2001), it appears to have additional functions in the context of malignancy (Ilantzis *et al*, 2002; Duxbury *et al*, 2004b). Despite lacking transmembrane and intracellular domains, this GPI-anchored protein is able to influence intracellular pathways central to cancer development and progression. We have recently reported that overexpression of CEACAM6 is associated with an increased ability of pancreatic cancer cells to survive under anchorage-independent conditions and that post-transcriptional silencing of CEACAM6 expression suppresses this ability, as well as impairing tumour progression in a murine pancreatic adenocarcinoma xenograft model (Duxbury *et al*, 2004b).

Glycosylphosphatidylinositol-anchored proteins have been recognised to modulate activity of intracellular tyrosine kinases (Stefanova *et al*, 1991; Brown, 1993; Skubitz *et al*, 1995) and our results indicate that c-Src, which plays a central role in pancreatic adenocarcinoma cellular invasion (Ito *et al*, 2003), may act as an effector of CEACAM6 signalling. The 92 kDa type IV collagenase, MMP-9, plays a critical role in physiological and pathological tissue remodelling. MMP-9 is transcriptionally regulated in a c-Src-dependent manner through a GT box located downstream of the AP-1 transcription factor-binding site of its promoter (Sato *et al*, 1993). Modulation of c-Src tyrosine kinase activity alters MMP-9 activity in a variety of tumour cell types, including pancreatic adenocarcinoma cells (Ito *et al*, 2003; Recchia *et al*, 2003). Our results are consistent with a model in which CEACAM6 overexpression leads to a c-Src-dependent increase in MMP-9 activity, resulting in increased cellular invasiveness. We have recently examined the mechanisms through which CEACAM6 modulates activity of c-Src kinase activity (Duxbury *et al*, 2004a). CEACAM6 appears to be coupled to the inhibitor of c-Src, Csk, in a caveolin-1 dependent manner. Antibody-mediated crosslinking of CEACAM6 results in a caveolin-1-dependent increase in c-Src activity in BxPC3 cells. The increased c-Src activity exhibited by pIRES-CEACAM6.1 and pIRES-CEACAM6.2 transfectants may reflect auto-aggregation of the GPI-linked protein within membrane lipid rafts, as occurs when other PGI-linked proteins are present at high density in the cell membrane. We speculate that other membrane-associated molecules may modulate CEACAM6 aggregation and signal transduction, although this aspect of CEACAM6 biology requires further study.

RNA interference offers an unrivalled opportunity to silence expression of individual genes with a high degree of specificity, allowing the roles of individual genes to be dissected (Shi, 2003). However, one of the limitations of RNAi is the requirement for transfection of cells with siRNA. Transfection efficiency varies widely among cell types and exogenous siRNA oligonucleotide-mediated RNAi is transient in nature, due to siRNA dilution and degradation. Retroviral vectors have become a popular means of transfecting cells with a high degree of efficiency. This technique relies on the generation of short hairpin RNA (shRNA), which is cleaved by intracellular nucleases to yield active siRNA. This technique allows induction of stable RNAi (Brummelkamp *et al*, 2002). Using this technique to silence CEACAM6 expression, we have shown that levels of CEACAM6 expression can markedly influence the invasive cellular phenotype. Retroviruses selectively infect dividing cells, making them a rational vehicle for directed–directed gene silencing (Logg and Kasahara, 2004). However, viral gene therapies have raised cause for concern following well-publicised incidences of toxicity and death (Raper *et al*, 2003). Although clinical evaluation of these techniques will need to continue with caution, *in vivo* studies to date have reported no signs of systemic toxicity (Brummelkamp *et al*, 2002).

In summary, levels of CEACAM6 expression can modulate the invasive phenotype in a c-Src-dependent manner, through alterations in cellular MMP-9 activity. These findings support the premise that targeting CEACAM6 may represent a rational strategy to attenuate the invasiveness of this malignancy. Novel strategies targeting CEACAM6 and its downstream pathways warrant further evaluation in the context of gastrointestinal malignancy.
